# *rac*-2-(2-Amino-4-oxo-4,5-dihydro-1,3-thia­zol-5-yl)-2-hydroxy­indane-1,3-dione

**DOI:** 10.1107/S1600536809025574

**Published:** 2009-07-18

**Authors:** Narsimha Reddy Penthala, Thirupathi Reddy Yerram Reddy, Sean Parkin, Peter A. Crooks

**Affiliations:** a Department of Pharmaceutical Sciences, College of Pharmacy, University of Kentucky, Lexington, KY 40536, USA; b Department of Chemistry, University of Kentucky, Lexington, KY 40506, USA

## Abstract

In the crystal of the title compound, C_12_H_8_N_2_O_4_S, mol­ecules are linked into chains by a series of inter­molecular O—H⋯O, N—H⋯O and N—H⋯N hydrogen bonds. The ninhydrin and amino­thia­zolidine units make a dihedral angle of 66.41 (3)°. The crystal structure indicates the presence of equimolar *R* and *S* enanti­omers in the crystal lattice, due to the presence of a chiral centre in the title compound.

## Related literature

The NADPH-dependent oxidase activity of 2-indol-3-yl- methyl­enequinuclidin-3-ols has been reported by Sekhar *et al.* (2003 ▶) and novel substituted (*Z*)-2-(*N*-benzyl­indol-3-ylmethyl­ene) quinuclidin-3-one and (*Z*)-(±)-2-(*N*-benzyl­indol-3-ylmethyl­ene) quinuclidin-3-ol derivatives have been identified as potent thermal sensitizing agents (Sonar *et al.*, 2007 ▶). The crystal structure and bond-length data for ninhydrin have been described by Medrud (1969 ▶) and Fun *et al.* (2009 ▶).
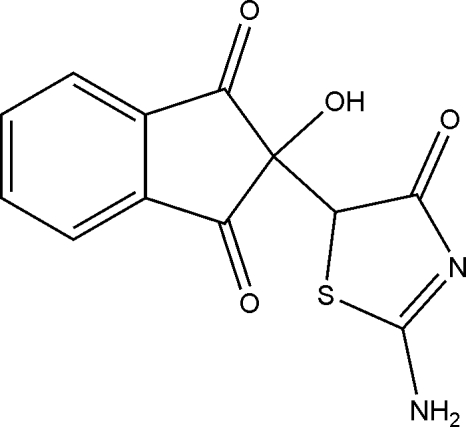


## Experimental

### 

#### Crystal data

C_12_H_8_N_2_O_4_S
*M* *_r_* = 276.26
Monoclinic, 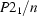

*a* = 14.1702 (2) Å
*b* = 5.6713 (1) Å
*c* = 14.8296 (3) Å
β = 114.0171 (9)°
*V* = 1088.58 (3) Å^3^
*Z* = 4
Mo *K*α radiation
μ = 0.31 mm^−1^
*T* = 90 K
0.25 × 0.12 × 0.05 mm

#### Data collection

Nonius KappaCCD diffractometer
Absorption correction: multi-scan (*SCALEPACK*; Otwinowski & Minor, 1997 ▶) *T*_min_ = 0.927, *T*_max_ = 0.985
23811 measured reflections
2498 independent reflections
2268 reflections with *I* > 2σ(*I*)
*R*_int_ = 0.035

#### Refinement

*R*[*F* ^2^ > 2σ(*F* ^2^)] = 0.029
*wR*(*F* ^2^) = 0.076
*S* = 1.06
2498 reflections
173 parameters
H-atom parameters constrained
Δρ_max_ = 0.38 e Å^−3^
Δρ_min_ = −0.26 e Å^−3^

### 

Data collection: *COLLECT* (Nonius, 1998 ▶); cell refinement: *SCALEPACK* (Otwinowski & Minor, 1997 ▶); data reduction: *DENZO-SMN* (Otwinowski & Minor, 1997 ▶); program(s) used to solve structure: *SHELXS97* (Sheldrick, 2008 ▶); program(s) used to refine structure: *SHELXL97* (Sheldrick, 2008 ▶); molecular graphics: *XP* in *SHELXTL* (Sheldrick, 2008 ▶); software used to prepare material for publication: *SHELXL97* and local procedures.

## Supplementary Material

Crystal structure: contains datablocks global, I. DOI: 10.1107/S1600536809025574/hg2529sup1.cif

Structure factors: contains datablocks I. DOI: 10.1107/S1600536809025574/hg2529Isup2.hkl

Additional supplementary materials:  crystallographic information; 3D view; checkCIF report

## Figures and Tables

**Table 1:** Hydrogen-bond geometry (Å, °)

*D*—H⋯*A*	*D*—H	H⋯*A*	*D*⋯*A*	*D*—H⋯*A*
O2—H2⋯O3^i^	0.84	1.99	2.8225 (13)	170
N2—H2*A*⋯N1^ii^	0.88	2.07	2.9372 (15)	168
N2—H2*B*⋯O2^iii^	0.88	2.14	2.9629 (14)	155

Symmetry codes: (i) 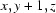; (ii) 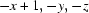; (iii) 
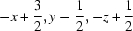
.

**Fig. 1.**
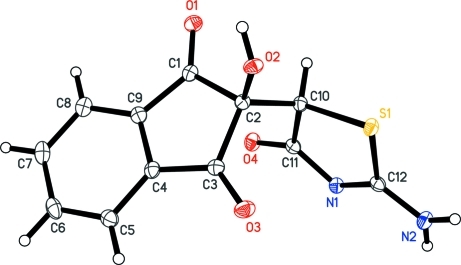
A view of the molecule with the atom numbering scheme. Displacement ellipsoids are drawn at the 50% probability level.

**Fig. 2.**
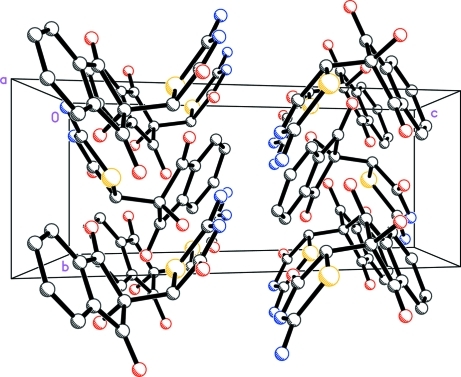
Crystal packing of the molecule viewed along the a axis. H atoms have been omitted for clarity.

## supplementary crystallographic information

### Comment

In continuing studies on the design and synthesis of novel radiosensitizers such as (*Z*)-2-(*N*-benzylindol-3-ylmethylene)quinuclidin-3-one and (*Z*)-(±)-2-(*N*-benzylindol-3-ylmethylene) quinuclidin-3-ol derivatives (Sekhar *et al.*, 2003; Sonar *et al.*, 2007), we have undertaken the design, synthesis and structural analysis of a series of ninhydrin analogs with a variety of active methylene compounds. The X-ray analysis of the title compound was carried out to confirm stereochemistry and to obtain detailed information on the structural conformation of the molecule, that might be useful in structure-activity relationship (SAR) studies. The title compound was prepared by the condensation of ninhydrin with 2-aminothiazol-4(5*H*)-one in ethanol at reflux temperature. The compound was crystallized from ethanol. The molecular structure and the atom-numbering scheme are shown in Fig.1. The ninhydrin ring is planar (r.m.s. deviation = 0.0255 (10) Å) with bond distances and angles comparable with those previously reported for ninhydrin (Medrud, 1969 and Fun *et al.* (2009). The title compound has a chiral centre at C_10_ and the X-ray data indicate that the compound is racemic (Fig. 2). The ninhydrin and 2-aminothiazol- 4(5*H*)-one moieties make a dihedral angle of 66.41 (3)°. Intermolecular O—H···O, N—H···O and N—H···N hydrogen bonds stabilize the crystal structure, and form a three-dimensional network.

### Experimental

A mixture of ninhydrin (1 mmol) and 2-aminothiazol-4(5*H*)-one (1.1 mmol) was stirred under reflux in ethanol for 6 hrs. After the reaction was complete, the reaction mixture was cooled to room temperature. The precipitate thus obtained was collected by filtration, washed with cold ethanol and dried, to afford the crude product. Crystallization from ethanol afforded a light yellow crystalline product of 2-(2-amino-4-oxo-4,5-dihydrothiazol-5-yl)-2-hydroxy -1*H*-indene-1,3(2*H*)-dione that was suitable for X-ray analysis. ^1^H NMR (DMSO-d_6_): δ 5.01 (*s*, 1H, CH), 7.10 (*s*, 1H, OH), 7.93–8.03 (*m*, 4H, Ar—H), 8.94–9.06 (*bd*, 2H, NH_2_), p.p.m.; ^13^C NMR (DMSO-d_6_): δ 62.15, 73.62, 123.47, 123.57, 136.67, 137.26, 140.68, 141.09, 182.49, 185.12, 197.48, 198.25 p.p.m..

### Refinement

H atoms were found in difference Fourier maps and subsequently placed in idealized positions with constrained distances of 1.00 Å (*R*~3~CH), 0.95 Å (CÃr~H), 0.84 Å (O—H), 0.88 Å (N—H), and with *U*ĩso~(H) values set to either 1.2*U*~eq~ or 1.5*U*~eq~(OH) of the attached atom.

### Crystal data

**Table:**

C_12_H_8_N_2_O_4_S	*F*(000) = 568
*M**_r_* = 276.26	*D*_x_ = 1.686 Mg m^−^^3^
Monoclinic, *P*2_1_/*n*	Mo *K*α radiation, λ = 0.71073 Å
Hall symbol: -P 2yn	Cell parameters from 2750 reflections
*a* = 14.1702 (2) Å	θ = 1.0–27.5°
*b* = 5.6713 (1) Å	µ = 0.31 mm^−^^1^
*c* = 14.8296 (3) Å	*T* = 90 K
β = 114.0171 (9)°	Tablet, pale yellow
*V* = 1088.58 (3) Å^3^	0.25 × 0.12 × 0.05 mm
*Z* = 4	

### Data collection

**Table:**

Nonius KappaCCD diffractometer	2498 independent reflections
Radiation source: fine-focus sealed tube	2268 reflections with *I* > 2σ(*I*)
graphite	*R*_int_ = 0.035
Detector resolution: 9.1 pixels mm^-1^	θ_max_ = 27.5°, θ_min_ = 1.7°
ω scans at fixed χ = 55°	*h* = −18→18
Absorption correction: multi-scan (*SCALEPACK*; Otwinowski & Minor, 1997)	*k* = −7→7
*T*_min_ = 0.927, *T*_max_ = 0.985	*l* = −19→19
23811 measured reflections	

### Refinement

**Table:**

Refinement on *F*^2^	Primary atom site location: structure-invariant direct methods
Least-squares matrix: full	Secondary atom site location: difference Fourier map
*R*[*F*^2^ > 2σ(*F*^2^)] = 0.029	Hydrogen site location: inferred from neighbouring sites
*wR*(*F*^2^) = 0.076	H-atom parameters constrained
*S* = 1.06	*w* = 1/[σ^2^(*F*_o_^2^) + (0.0342*P*)^2^ + 0.6796*P*], where *P* = (*F*_o_^2^ + 2*F*_c_^2^)/3
2498 reflections	(Δ/σ)_max_ < 0.001
173 parameters	Δρ_max_ = 0.38 e Å^−^^3^
0 restraints	Δρ_min_ = −0.26 e Å^−^^3^

### Special details

**Table:**

Geometry. All e.s.d.'s (except the e.s.d. in the dihedral angle between two l.s. planes) are estimated using the full covariance matrix. The cell e.s.d.'s are taken into account individually in the estimation of e.s.d.'s in distances, angles and torsion angles; correlations between e.s.d.'s in cell parameters are only used when they are defined by crystal symmetry. An approximate (isotropic) treatment of cell e.s.d.'s is used for estimating e.s.d.'s involving l.s. planes.
Refinement. Refinement of *F*^2^ against ALL reflections. The weighted *R*-factor *wR* and goodness of fit *S* are based on *F*^2^, conventional *R*-factors *R* are based on *F*, with *F* set to zero for negative *F*^2^. The threshold expression of *F*^2^ > 2σ(*F*^2^) is used only for calculating *R*-factors(gt) *etc*. and is not relevant to the choice of reflections for refinement. *R*-factors based on *F*^2^ are statistically about twice as large as those based on *F*, and *R*- factors based on ALL data will be even larger.

### Fractional atomic coordinates and isotropic or equivalent isotropic displacement parameters (Å^2^)

**Table:**

	*x*	*y*	*z*	*U*_iso_*/*U*_eq_	
S1	0.66382 (2)	0.49412 (5)	0.17138 (2)	0.01284 (10)	
O1	0.42723 (7)	0.96668 (16)	0.23512 (7)	0.0153 (2)	
N1	0.49352 (8)	0.24974 (19)	0.06881 (8)	0.0125 (2)	
C1	0.45157 (9)	0.7729 (2)	0.27136 (9)	0.0120 (2)	
O2	0.63896 (6)	0.76852 (16)	0.33559 (7)	0.01299 (19)	
H2	0.6250	0.9129	0.3335	0.019*	
N2	0.64515 (8)	0.0977 (2)	0.06834 (8)	0.0143 (2)	
H2A	0.6112	−0.0200	0.0303	0.017*	
H2B	0.7125	0.1084	0.0878	0.017*	
C2	0.54859 (9)	0.6411 (2)	0.27535 (9)	0.0111 (2)	
O3	0.61330 (7)	0.25829 (17)	0.35097 (7)	0.0167 (2)	
C3	0.54791 (9)	0.4118 (2)	0.33094 (9)	0.0120 (2)	
O4	0.37082 (7)	0.45803 (17)	0.10054 (7)	0.0162 (2)	
C4	0.45890 (10)	0.4216 (2)	0.35841 (9)	0.0127 (2)	
C5	0.42889 (10)	0.2577 (2)	0.41132 (9)	0.0153 (3)	
H5	0.4654	0.1135	0.4326	0.018*	
C6	0.34424 (10)	0.3100 (3)	0.43217 (9)	0.0178 (3)	
H6	0.3220	0.1996	0.4676	0.021*	
C7	0.29109 (10)	0.5234 (3)	0.40164 (10)	0.0180 (3)	
H7	0.2348	0.5578	0.4186	0.022*	
C8	0.31936 (10)	0.6860 (3)	0.34679 (9)	0.0158 (3)	
H8	0.2823	0.8293	0.3248	0.019*	
C9	0.40390 (9)	0.6314 (2)	0.32529 (9)	0.0130 (2)	
C10	0.54450 (9)	0.6061 (2)	0.17129 (9)	0.0116 (2)	
H10	0.5296	0.7620	0.1368	0.014*	
C11	0.45938 (9)	0.4304 (2)	0.10930 (9)	0.0121 (2)	
C12	0.59513 (9)	0.2577 (2)	0.09614 (9)	0.0121 (2)	

### Atomic displacement parameters (Å^2^)

**Table:**

	*U*^11^	*U*^22^	*U*^33^	*U*^12^	*U*^13^	*U*^23^
S1	0.01022 (15)	0.01467 (17)	0.01409 (16)	−0.00198 (11)	0.00543 (12)	−0.00411 (11)
O1	0.0149 (4)	0.0140 (5)	0.0158 (4)	0.0023 (4)	0.0051 (4)	0.0012 (4)
N1	0.0116 (5)	0.0137 (5)	0.0120 (5)	−0.0008 (4)	0.0046 (4)	−0.0010 (4)
C1	0.0101 (5)	0.0138 (6)	0.0107 (5)	−0.0008 (5)	0.0027 (4)	−0.0026 (5)
O2	0.0108 (4)	0.0104 (4)	0.0154 (4)	−0.0007 (3)	0.0030 (3)	−0.0023 (3)
N2	0.0109 (5)	0.0154 (5)	0.0162 (5)	−0.0014 (4)	0.0051 (4)	−0.0049 (4)
C2	0.0098 (5)	0.0113 (6)	0.0117 (5)	−0.0005 (4)	0.0038 (4)	−0.0009 (5)
O3	0.0153 (4)	0.0131 (5)	0.0209 (5)	0.0027 (4)	0.0065 (4)	0.0013 (4)
C3	0.0123 (6)	0.0118 (6)	0.0101 (5)	−0.0012 (5)	0.0029 (5)	−0.0015 (5)
O4	0.0112 (4)	0.0197 (5)	0.0176 (4)	0.0001 (4)	0.0056 (4)	−0.0015 (4)
C4	0.0133 (6)	0.0139 (6)	0.0108 (6)	−0.0017 (5)	0.0047 (5)	−0.0029 (5)
C5	0.0180 (6)	0.0150 (6)	0.0125 (6)	−0.0023 (5)	0.0056 (5)	−0.0005 (5)
C6	0.0193 (6)	0.0234 (7)	0.0113 (6)	−0.0065 (5)	0.0071 (5)	−0.0021 (5)
C7	0.0131 (6)	0.0278 (8)	0.0136 (6)	−0.0039 (5)	0.0060 (5)	−0.0046 (5)
C8	0.0122 (6)	0.0199 (7)	0.0140 (6)	−0.0001 (5)	0.0041 (5)	−0.0026 (5)
C9	0.0123 (6)	0.0146 (6)	0.0109 (5)	−0.0022 (5)	0.0035 (5)	−0.0017 (5)
C10	0.0109 (5)	0.0125 (6)	0.0119 (5)	−0.0002 (4)	0.0051 (4)	−0.0006 (5)
C11	0.0126 (6)	0.0133 (6)	0.0095 (5)	−0.0006 (5)	0.0035 (5)	0.0009 (5)
C12	0.0137 (6)	0.0128 (6)	0.0096 (5)	−0.0017 (5)	0.0046 (4)	0.0004 (5)

### Geometric parameters (Å, °)

**Table:**

S1—C12	1.7623 (13)	C3—C4	1.4759 (17)
S1—C10	1.8056 (12)	O4—C11	1.2180 (16)
O1—C1	1.2101 (16)	C4—C5	1.3903 (18)
N1—C12	1.3283 (16)	C4—C9	1.3973 (18)
N1—C11	1.3715 (17)	C5—C6	1.3873 (19)
C1—C9	1.4764 (17)	C5—H5	0.9500
C1—C2	1.5449 (17)	C6—C7	1.400 (2)
O2—C2	1.4245 (14)	C6—H6	0.9500
O2—H2	0.8400	C7—C8	1.3922 (19)
N2—C12	1.3166 (16)	C7—H7	0.9500
N2—H2A	0.8800	C8—C9	1.3942 (18)
N2—H2B	0.8800	C8—H8	0.9500
C2—C10	1.5334 (17)	C10—C11	1.5460 (17)
C2—C3	1.5418 (17)	C10—H10	1.0000
O3—C3	1.2167 (16)		
			
C12—S1—C10	89.51 (6)	C5—C6—C7	120.88 (12)
C12—N1—C11	111.88 (11)	C5—C6—H6	119.6
O1—C1—C9	128.58 (12)	C7—C6—H6	119.6
O1—C1—C2	123.02 (11)	C8—C7—C6	121.13 (12)
C9—C1—C2	108.20 (10)	C8—C7—H7	119.4
C2—O2—H2	109.5	C6—C7—H7	119.4
C12—N2—H2A	120.0	C7—C8—C9	117.69 (13)
C12—N2—H2B	120.0	C7—C8—H8	121.2
H2A—N2—H2B	120.0	C9—C8—H8	121.2
O2—C2—C10	110.66 (10)	C8—C9—C4	121.14 (12)
O2—C2—C3	107.00 (9)	C8—C9—C1	128.92 (12)
C10—C2—C3	115.04 (10)	C4—C9—C1	109.91 (11)
O2—C2—C1	109.71 (10)	C2—C10—C11	112.48 (10)
C10—C2—C1	110.82 (10)	C2—C10—S1	113.13 (8)
C3—C2—C1	103.28 (10)	C11—C10—S1	106.11 (8)
O3—C3—C4	127.68 (12)	C2—C10—H10	108.3
O3—C3—C2	124.33 (11)	C11—C10—H10	108.3
C4—C3—C2	107.91 (10)	S1—C10—H10	108.3
C5—C4—C9	120.86 (12)	O4—C11—N1	125.52 (12)
C5—C4—C3	128.50 (12)	O4—C11—C10	120.08 (11)
C9—C4—C3	110.63 (11)	N1—C11—C10	114.40 (10)
C6—C5—C4	118.26 (12)	N2—C12—N1	122.39 (12)
C6—C5—H5	120.9	N2—C12—S1	119.58 (9)
C4—C5—H5	120.9	N1—C12—S1	118.02 (10)
			
O1—C1—C2—O2	64.08 (15)	C5—C4—C9—C1	−179.91 (11)
C9—C1—C2—O2	−111.21 (11)	C3—C4—C9—C1	1.32 (14)
O1—C1—C2—C10	−58.41 (15)	O1—C1—C9—C8	0.5 (2)
C9—C1—C2—C10	126.30 (11)	C2—C1—C9—C8	175.40 (12)
O1—C1—C2—C3	177.88 (11)	O1—C1—C9—C4	−177.45 (12)
C9—C1—C2—C3	2.59 (12)	C2—C1—C9—C4	−2.51 (14)
O2—C2—C3—O3	−63.22 (15)	O2—C2—C10—C11	169.35 (10)
C10—C2—C3—O3	60.14 (16)	C3—C2—C10—C11	47.95 (14)
C1—C2—C3—O3	−178.97 (12)	C1—C2—C10—C11	−68.72 (13)
O2—C2—C3—C4	113.91 (10)	O2—C2—C10—S1	49.13 (12)
C10—C2—C3—C4	−122.72 (11)	C3—C2—C10—S1	−72.27 (12)
C1—C2—C3—C4	−1.84 (12)	C1—C2—C10—S1	171.06 (8)
O3—C3—C4—C5	−1.2 (2)	C12—S1—C10—C2	124.72 (9)
C2—C3—C4—C5	−178.24 (12)	C12—S1—C10—C11	0.93 (9)
O3—C3—C4—C9	177.42 (12)	C12—N1—C11—O4	−176.05 (12)
C2—C3—C4—C9	0.41 (14)	C12—N1—C11—C10	3.27 (15)
C9—C4—C5—C6	−1.35 (18)	C2—C10—C11—O4	52.60 (16)
C3—C4—C5—C6	177.19 (12)	S1—C10—C11—O4	176.80 (10)
C4—C5—C6—C7	−0.65 (19)	C2—C10—C11—N1	−126.76 (11)
C5—C6—C7—C8	2.1 (2)	S1—C10—C11—N1	−2.56 (13)
C6—C7—C8—C9	−1.44 (19)	C11—N1—C12—N2	178.04 (12)
C7—C8—C9—C4	−0.56 (19)	C11—N1—C12—S1	−2.57 (14)
C7—C8—C9—C1	−178.27 (12)	C10—S1—C12—N2	−179.74 (11)
C5—C4—C9—C8	1.99 (19)	C10—S1—C12—N1	0.85 (10)
C3—C4—C9—C8	−176.79 (11)		

### Hydrogen-bond geometry (Å, °)

**Table:**

*D*—H···*A*	*D*—H	H···*A*	*D*···*A*	*D*—H···*A*
O2—H2···O3^i^	0.84	1.99	2.8225 (13)	170
N2—H2A···N1^ii^	0.88	2.07	2.9372 (15)	168
N2—H2B···O2^iii^	0.88	2.14	2.9629 (14)	155

Symmetry codes: (i) *x*, *y*+1, *z*; (ii) −*x*+1, −*y*, −*z*; (iii) −*x*+3/2, *y*−1/2, −*z*+1/2.
